# Provenance, Not Prohibition: A Framework for AI in Scholarly Publishing

**DOI:** 10.1002/lio2.70487

**Published:** 2026-06-29

**Authors:** Romaine F. Johnson

**Affiliations:** ^1^ UT Southwestern Dallas Texas USA

**Keywords:** disclosure, generative artificial intelligence, peer review, provenance, research integrity, scholarly publishing

## Abstract

Generative AI is now embedded in scholarly workflows, yet publishing policy has often jumped to permission or prohibition without first defining what is being governed. The central risk is not tool use itself, but loss of provenance: readers, reviewers, and editors must be able to trace how claims, citations, analyses, and interpretations were produced and verified. Harm is already measurable, including fabricated references, distorted summaries, hollow reviews, and disclosure policies that are widely ignored. This commentary proposes a practical framework centered on human accountability: separate capability from responsibility, prioritize provenance over polish, avoid unverifiable bans, and pair disclosure with explicit verification. Because AI now enters every stage of review, the unit of governance is not the static manuscript but the feedback loop around it, and the same obligations bind authors, reviewers, and editors alike. Journals should govern outcomes and professional standards rather than tool lists, building a culture of transparent use instead of surveillance.

Every surgical discipline teaches the same first lesson: know what you are looking at before deciding what to do. The conversation around artificial intelligence in scholarly publishing has largely skipped that step. We have moved directly to policy—banning, permitting, requiring disclosure—without first establishing a shared understanding of what we are governing and why it matters. Generative AI is now part of how scholarship is written. That expands access and improves efficiency, but it also creates a deficit of provenance. The right question is not whether AI belongs in our workflows, but how we preserve accountability when it does.

The risks are concrete and documented. In one systematic evaluation, 18%–55% of AI‐generated bibliographic citations were entirely fabricated: plausible‐looking references to papers that do not exist [1]. Language models distort the conclusions of studies they summarize and can expose confidential patient or institutional data when users paste unpublished material into commercial platforms. In peer review, AI‐generated assessments have appeared that sound competent but lack the disciplinary judgment that makes review meaningful. That harm begins on the reviewer's side of the desk, not the author's. Current policies are failing on their own terms. An analysis of 5.2 million papers found that only 0.1% disclosed AI use even though most journals had formal disclosure requirements; adoption rates did not differ between journals with and without such policies [2]. These are failures of implementation, not evidence that AI and scholarship are incompatible. Six principles follow, offered less as rules than as a framework for examining our institutional reflexes before they harden into policy that addresses the wrong problem.

**Separate capability from accountability**. A language model can generate a lucid methods section describing mixed‐effects regression with appropriate covariate justification. It can also explain why a negative binomial distribution fits overdispersed count data better than Poisson, and increasingly it can defend such choices when asked. The capability line has moved, and candor requires saying so. What a model cannot do is answer for the choice. It cannot stand at the podium as the person whose license, reputation, and standing are pledged to it, be cross‐examined on it, and be held to a consistent position across years and consequences. Authorship has never been about who produced the words, or even the reasoning. It has always been about who can answer for them. The senior author on a multicenter trial did not personally pipette every sample or interview every patient. What makes that author accountable is the obligation to interrogate every decision in the chain. AI can produce scholarship, and now its rationale. Only a human can answer for it.

**Provenance beats perfection**. In clinical medicine, we trust the surgeon who says, “I encountered unexpected findings and adapted,” more than the one whose operative note reads like a textbook. The same logic applies to scholarship. Readers do not merely evaluate claims; they evaluate the process that produced them. A manuscript with transparent methodology invites productive critique. An opaque one, however polished, breeds suspicion. When we cannot trace how knowledge was constructed, we lose the ability to build on it, challenge it, or correct it. Provenance is not a bureaucratic requirement. It is the epistemic infrastructure of science.

**Do not build policy around prohibitions you cannot verify**. AI detection tools remain unreliable, particularly for scientific prose, which is already standardized by convention. A well‐written methods section and an AI‐generated one can be linguistically indistinguishable, not because the AI is deceptive, but because rigorous scientific writing is formulaic by design. A bibliometric analysis of the top 100 publishers confirmed that most permit AI‐assisted writing, yet their guidelines varied so widely that some journals' policies directly contradicted their publishers' own positions [3]. Prohibition creates a perverse incentive structure: the author who discloses AI assistance for language polishing faces scrutiny, while the one who conceals its use in generating analytic interpretations goes undetected. That asymmetry matters. Using AI to refine grammar differs categorically from using it to draft a discussion section or select a statistical model. Policy should recognize that gradient rather than treat all AI use as ethically equivalent.

**Pair disclosure with verification**. A simple attestation—“AI tools were used in manuscript preparation; all citations, data, and analyses were independently verified by the authors”—does more than any detection algorithm. This is professional diligence, no different from declaring conflicts of interest or confirming IRB approval. Recent consensus work, including a modified Delphi study of authors, reviewers, editors, and AI researchers, has converged on this framework: transparency, human accountability, and protection of confidential material as organizing principles rather than tool‐specific prohibitions [4, 5]. The attestation reframes the question from *whether* AI was used to *whether scholarly standards were met*. The mechanism matters. Bare disclosure collapsed to near zero because it was a costless checkbox, detached from any substantive claim. An attestation instead binds disclosure to verification, as a conflict‐of‐interest declaration or IRB confirmation does, by asserting something a colleague can hold the signer to. The obligation is symmetric. A reviewer who drafts a critique with a model, or an editor who synthesizes one, owes the same attestation, especially because the disciplinary judgment a review is meant to supply is exactly what a model cannot vouch for. For journals with international author bases, where English may be a third language and institutional resources vary widely, this approach has an additional virtue: it broadens access to polished scientific communication without pretending the tools do not exist.

**Govern outcomes, not tool lists**. As AI becomes ambient, embedded in word processors, reference managers, and statistical software, policing specific applications becomes an exercise in futility. We do not ask surgeons to disclose which brand of electrocautery they used; we ask whether the operation was performed competently and whether complications were managed honestly. The meaningful question is whether the author can defend the thesis, explain the methodology, and engage criticism. One carve‐out is real, however. Confidentiality cannot be policed after the fact: once a colleague pastes an unpublished manuscript into a commercial platform, the breach is complete and invisible. Protecting confidential material is a front‐end control, a matter of where text is processed, and the one place where the tool itself is a legitimate object of policy. Everywhere else, accountability remains human even as the instruments evolve.

**Build culture, not surveillance**. Academic integrity has never been sustained primarily by detection. It has been sustained by shared values, modeled behavior, and the understanding that trust, once lost, is expensive to rebuild. When senior scholars and editors model thoughtful AI use and transparent disclosure, the next generation of investigators follows. When we treat AI as a shameful secret rather than a tool that requires judgment, we guarantee misuse.

These six principles share a common architecture: they shift accountability from the tool to the human, from the input to the output, and from prohibition to transparency. They also shift the unit of governance. We are accustomed to treating the manuscript as the thing to be certified: a static artifact, inspected and stamped. But AI now enters every stage of the exchange around it. The author drafts with a model, the reviewer critiques with one, the author rebuts with one, and the editor adjudicates with one. Provenance does not live in the finished file. It lives in that correspondence, where trust is built or lost. The obligations described here bind everyone in the loop—author, reviewer, and editor—or they bind no one credibly.

Principles without a standard remain aspirational. Journals do not need exhaustive catalogs of permissible and impermissible software. They need a clear professional expectation, owed by every participant in review: AI may be used in preparing a manuscript, a critique, or a decision, but whoever uses it must disclose that use, independently verify all citations, data, and analyses, protect confidential material, and remain fully accountable for every claim advanced under their name. The tools will change quickly. Human responsibility will not.

## Funding

The author has nothing to report.

## Ethics Statement

The author has nothing to report.

## Consent

The author has nothing to report.

## Conflicts of Interest

Dr. Romaine F. Johnson is the Editor in Chief of Laryngoscope Investigative Otolaryngology.

## Data Availability

Data sharing is not applicable to this article as no new data were created or analyzed in this study.
